# Phenotypic divergence in an island bee population: Applying geometric morphometrics to discriminate population‐level variation in wing venation

**DOI:** 10.1002/ece3.10085

**Published:** 2023-05-10

**Authors:** Madeleine M. Ostwald, Charles N. Thrift, Katja C. Seltmann

**Affiliations:** ^1^ Cheadle Center for Biodiversity and Ecological Restoration University of California Santa Barbara Santa Barbara California USA

**Keywords:** California Channel Islands, *Halictus tripartitus*, island‐mainland variation, wing morphology

## Abstract

Phenotypic divergence is an important consequence of restricted gene flow in insular populations. This divergence can be challenging to detect when it occurs through subtle shifts in morphological traits, particularly in traits with complex geometries, like insect wing venation. Here, we employed geometric morphometrics to assess the extent of variation in wing venation patterns across reproductively isolated populations of the social sweat bee, *Halictus tripartitus*. We examined wing morphology of specimens sampled from a reproductively isolated population of *H. tripartitus* on Santa Cruz Island (Channel Islands, Southern California). Our analysis revealed significant differentiation in wing venation in this island population relative to conspecific mainland populations. We additionally found that this population‐level variation was less pronounced than the species‐level variation in wing venation among three sympatric congeners native to the region, *Halictus tripartitus*, *Halictus ligatus*, and *Halictus farinosus*. Together, these results provide evidence for subtle phenotypic divergence in an island bee population. More broadly, these results emphasize the utility and potential of wing morphometrics for large‐scale assessment of insect population structure.

## Graphical abstract

We used geometric morphometrics to demonstrate phenotypic divergence in island and mainland populations of the sweat bee, *Halictus tripartitus*. The two populations show subtle morphological differentiation in the venation patterns on their forewings.

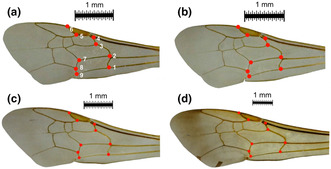

## 1 INTRODUCTION

Insular conditions are major drivers of population‐level phenotypic differentiation (Meröndun et al., 2019; Phillimore et al., 2008; Runemark et al., 2014; Velo‐Antón & Cordero‐Rivera, 2017). In particular, island populations can experience rapid evolutionary changes in morphological traits due to founder effect and subsequent genetic drift (Alsos et al., 2015; Barton, 1996; Hedrick et al., 2001; Jordan & Snell, 2008; Santos de Souza et al., 2019; Sylvester et al., 1998; Velo‐Antón et al., 2012). As such, islands have long been considered natural testbeds for evolutionary questions (MacArthur & Wilson, 1963; Warren et al., 2015), and have illuminated patterns of morphological variation, especially among birds and mammals (Cooper & Purvis, 2010; Grant, 1965; Millien, 2006). In contrast, trait variation in island populations of insects remains relatively unexplored, despite the prominence of these systems as ecologically important pollinators, pests, and invasive species (Fortuna et al., 2022; Hölldobler & Wilson, 1990; Klein et al., 2007; Traveset et al., 2013).

Beyond these taxonomic biases, our understanding of phenotypic variation across populations is biased toward traits that are easily distinguished or quantified by human observers, such as body size and coloration (Doucet et al., 2004; Kraemer et al., 2019; Lomolino, 1985; Palkovacs, 2003). In contrast, variation in traits that present measurement challenges, such as morphological traits with complex geometries, tends to be underexplored. One such trait is the pattern of venation in insect wings. Veins provide the primary structural support for wings, and while the functional significance of variation in venation patterns remains largely unclear (Combes & Daniel, 2003), they are highly conserved in insect lineages and thus are useful in phylogenetic reconstructions and taxonomic determinations (Comstock & Needham, 1898; Sharkey & Roy, 2002). Indeed, many identifying characteristics in bee taxonomy are found in wing venation patterns, with characteristic variation distinguishing genera and species (Michener, 1994).

Within a species, however, wing venation may present subtler patterns of variation that are undetectable via traditional observation methods. Geometric morphometrics, a set of methods that allows for spatial analysis of biological forms, has emerged as a promising approach to quantifying variation in complex morphological traits (Mitteroecker & Gunz, 2009; Rohlf & Marcus, 1993). This approach has been successfully implemented to discriminate patterns of insect wing venation among (Baylac et al., 2003; Deregnaucourt et al., 2021; Francoy et al., 2009, 2012; Kaba et al., 2017; Perrard et al., 2014; Rattanawannee et al., 2010, 2015; Santoso et al., 2018; Villemant et al., 2007) and even within species (Francisco et al., 2008; Francoy et al., 2011, 2016). Geometric morphometrics, therefore, has potential to assess the extent of phenotypic divergence among discrete insect populations by quantifying variation in this highly conserved trait.

We examined trait variation among island and mainland native bee populations in a Southern California coastal ecoregion. Santa Cruz Island is a 249 km^2^ Pacific island located 32 km due south of mainland Santa Barbara, California. It is the largest of the California Channel Islands, an eight‐island archipelago notable for its biodiversity and endemic species and which has served as a site for many microevolutionary studies of island‐mainland variation (O'Reilly & Horn, 2004). Santa Cruz Island shares many of its bee fauna with mainland Santa Barbara (Seltmann, 2019), but the distance separating these locations generally precludes gene flow between populations. Bees typically forage within a few kilometers of their nesting sites, and dispersal distances are generally well under the 30 km water barrier separating Santa Cruz Island from the mainland (O'Reilly & Horn, 2004). Further, while stem‐ and wood‐nesting bees have heightened island dispersal capabilities due to human transport of wood materials (Poulsen & Rasmussen, 2020), ground‐nesting bees have limited opportunities for human‐mediated island dispersal. Honey bees (*Apis mellifera* Linnaeus, 1758) were eradicated from the island by 2004 and have not been observed there since (Naughton et al., 2014; Seltmann et al., 2019; Wenner et al., 2009), suggesting that the channel is not easily crossed even by medium‐sized bees. As such, we are confident that gene flow between island and mainland bee populations in this context is minimal to nonexistent, increasing the likelihood of phenotypic divergence between populations.

In this study, we investigate variation in wing venation in island and mainland populations of the sweat bee, *Halictus tripartitus* Cockerell, 1985. *H. tripartitus* is a widespread, ground‐nesting social bee native to western North America and locally abundant both in mainland Santa Barbara and on Santa Cruz Island. We analyze museum specimens using a geometric morphometrics framework to assess the extent of variation in wing venation patterns between these two reproductively isolated populations. To contextualize the degree of variation, we additionally characterize variation in wing venation between *H. tripartitus* and two sympatric congeners, *H. ligatus* Say, 1837 and *H. farinosus* Smith, 1853. In doing so, we assess the role of reproductive isolation on population differentiation of morphological traits.

## 2 METHODS

### 2.1 Specimens and wing imaging

**FIGURE 1:**
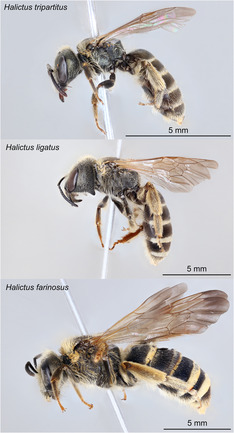
Lateral images of female *Halictus tripartitus* (top; UCSB‐IZC00040597), *H. ligatus* (middle; UCSB‐IZC00044094), and *H. farinosus* (bottom; UCSB‐IZC00042935) specimens. Images produced by Luz Ceja.

To assess population‐level variation in wing venation patterns, we imaged wings from three *Halictus* species: *H. tripartitus* (*n*_*island*_ = 149; *n*_*mainland*_ = 149), *H. ligatus* (*n*_*mainland*_ = 43), and *H. farinosus* (*n*_*island*_ = 3; *n*_*mainland*_ = 40; Figure 1). To achieve even sampling across species, we randomly selected 43 specimens of each species to analyze in our species‐level comparison. We obtained bee specimens from the University of California, Santa Barbara Invertebrate Zoology Collection housed by the Cheadle Center for Biodiversity and Ecological Restoration. All specimens were female and were collected between 1956 and 2020, with the majority of specimens collected recently (mean: 2018, median: 2019; Figure 2); (specimen catalog numbers available in Supplementary Materials). Species‐level identifications were confirmed by California native bee taxonomist Jaime Pawelek.

**FIGURE 2:**
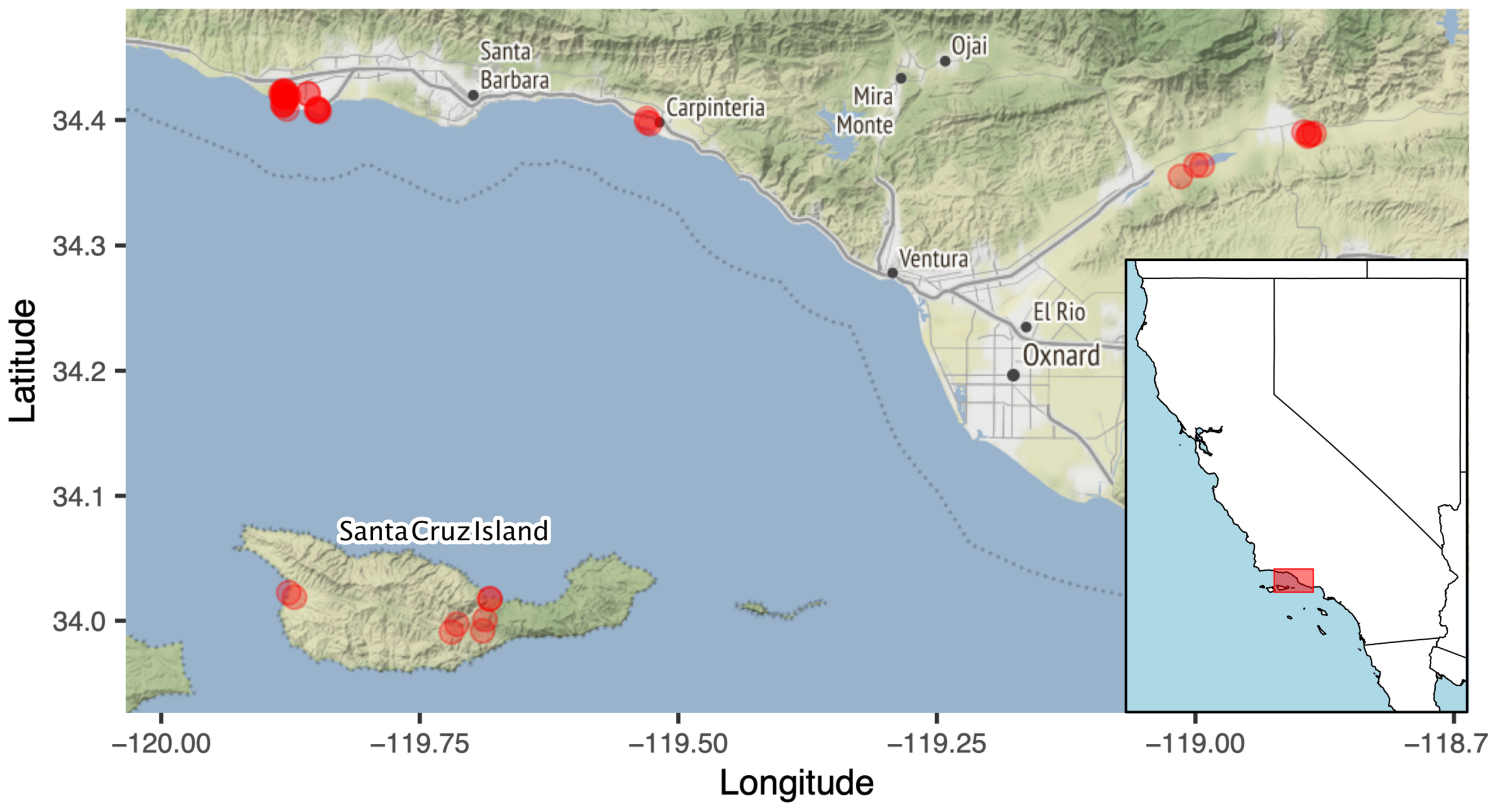
Map of sampling sites (red dots) on Santa Cruz Island and mainland Santa Barbara County and Ventura County. Inset map shows sampling region within California. *Halictus ligatus* was collected only on the mainland. *H. tripartitus* and *H. farinosus* were collected both on the mainland and on Santa Cruz Island.

**FIGURE 3:**
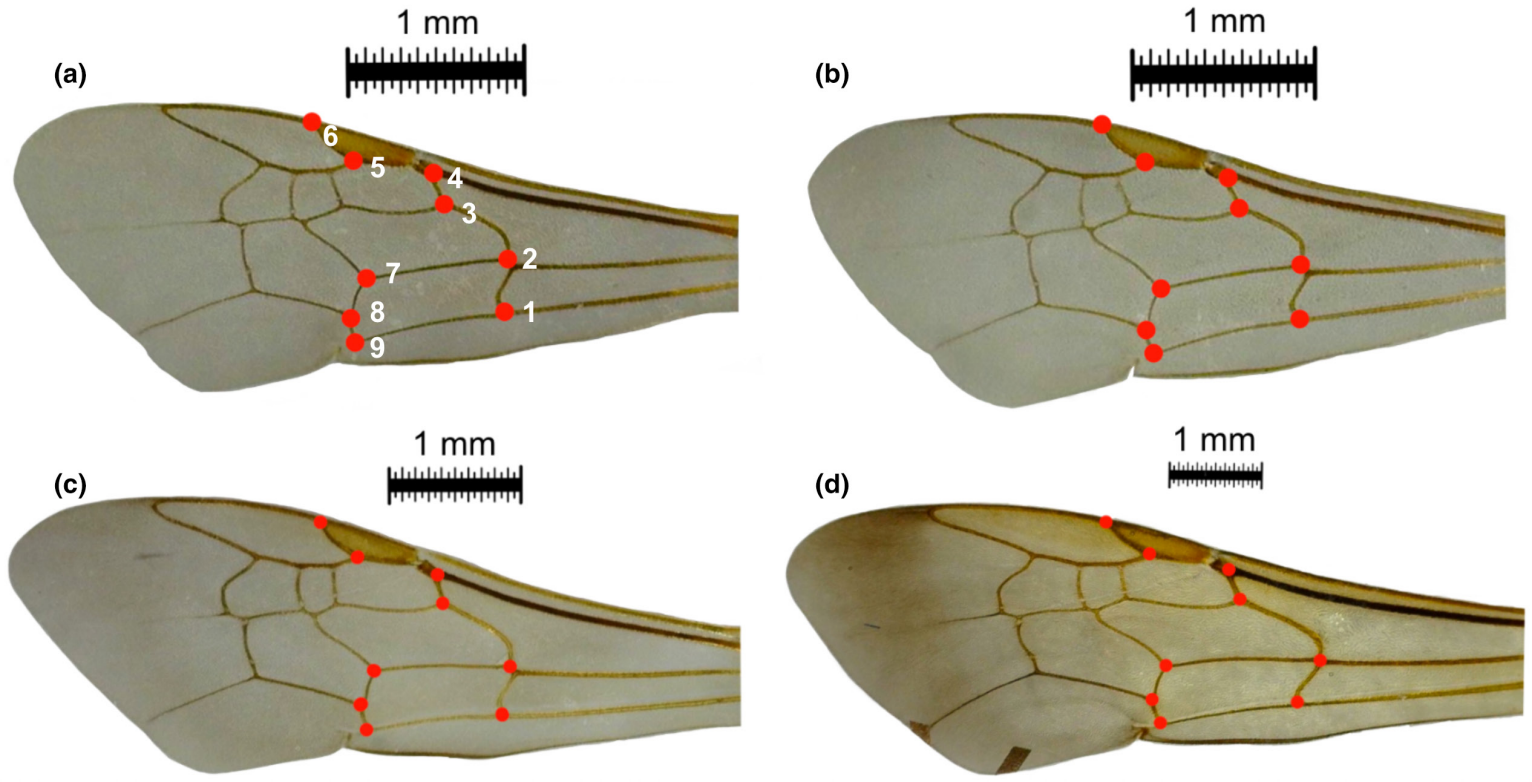
Landmarked images of *Halictus* forewings, including: (a) *Halictus tripartitus* from the mainland (UCSB‐IZC00042220), (b) *H. tripartitus* from Santa Cruz Island (UCSB‐IZC00040420), (c) *H. ligatus* (UCSB‐IZC00039120; mainland), and (d) *H. farinosus* (UCSB‐IZC00036955; Santa Cruz Island).

We removed left forewings from all specimens and imaged them with a stereo microscope digital camera along with a 1 mm scale slide (Dino‐Lite AM3111T; DinoXcope software 2.0.1). The basal tip of some forewings were removed if they were heavily sclerotized and prevented the wings from lying flat. We plotted 9 homologous wing venation landmarks (following Rattanawannee et al., 2015) onto each wing image using tpsDig software version 2.31 (Rohlf, 2015; Figure 3). All analysis was conducted in R version 4.2.2.

### 2.2 Data analysis

We Procrustes‐aligned landmark coordinates using R package “geomorph” version 4.0.0 (Adams et al., 2022; Baken et al., 2021). To test for statistical differences between the two *H. tripartitus* populations and among the three species, we ran one‐way multivariate analysis of variance (MANOVA) tests using R package “RRPP” version 1.3.1 (Collyer & Adams, 2018, 2019). To visualize separation among groups, we generated density plots with discriminant analysis of principal components (DAPC) using R package “adegenet” version 2.1.10 (Jombart, 2008; Jombart & Ahmed, 2011). To test the accuracy of using wing landmarks to predict an unknown bee's species or population, we utilized DAPC cross‐validation. Cross‐validation also informed the number of principal components (PCs) retained in each analysis (Jombart & Collins, 2015).

## 3 RESULTS

**FIGURE 4:**
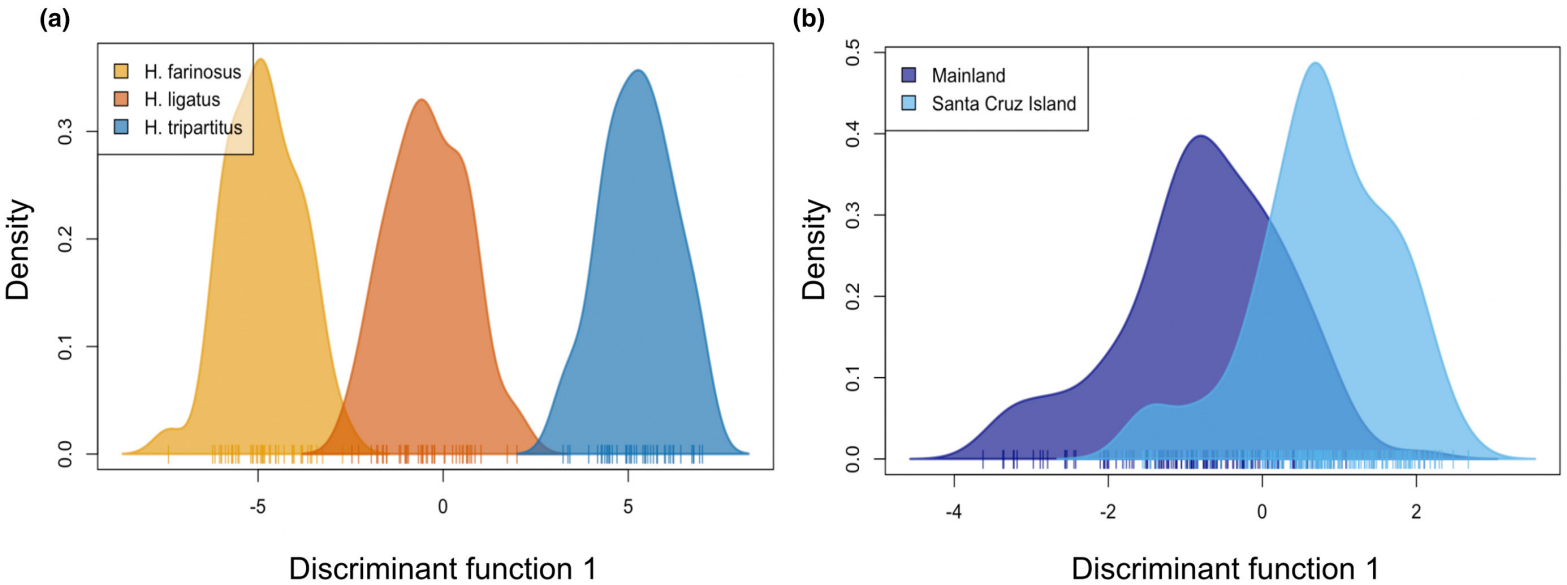
Density plots showing the most influential principal component for nine measured landmarks, comparing (a) three congeneric species of *Halictus* and (b) two separated populations of *H. tripartitus*.

Our analysis of wing landmark coordinates successfully discriminated between wings of *H. tripartitus*, *H. ligatus*, and *H. farinosus* (MANOVA: *Pillai* = 1.817, *p* < .001); (full MANOVA tables in Table 1; landmark coordinates in Table S1). Based on cross‐validation, 6 PCs were retained, and the density plot shows separation between species (Figure 4a). The cross‐validation test assigned 100% of *Halictus* specimens to their correct species (Figure S1a).

**TABLE 1:** MANOVA tables showing results of the comparison between three congeneric species of *Halictus* and two populations (island vs. mainland) of *H. tripartitus*.

Level of comparison	df	Residuals	Pillai	*Z*	Pr(>Pillai)
Between Species (*H. tripartitus*, *H. ligatus*, *H. farinosus*)	2	126	1.817	12.925	<.001
Within Species (Island vs. Mainland *H. tripartitus*)	1	296	0.425	7.715	<.001

Population‐level discrimination was also successful. The two populations of *H. tripartitus* differed significantly in wing landmark coordinates (MANOVA: *Pillai* = 0.425, *p* < .001; Table 1). Based on cross‐validation, 13 PCs were retained. The density plot shows some separation between populations, with overlap (Figure 4b). The cross‐validation test assigned 80.7% of *H. tripartitus* specimens to their correct population (Figure S1b). This analysis accurately identified the two subgroups (populations) of *H. tripartitus*, although with a lower degree of separation (MANOVA, DAPC) and accuracy (cross‐validation) than for the three congeneric *Halictus* species.

## 4 DISCUSSION

We employed a geometric morphometrics framework to demonstrate strong differentiation in wing venation geometries across three sympatric species of *Halictus* bees, as well as significant (though less pronounced) differentiation across island and mainland populations of *H. tripartitus*. Species‐level variation in wing venation is well established for many bee taxa, serving as useful characters for identification to family, genus, or species levels (Michener, 1994). Several studies have even successfully discriminated between distinct insect subspecies or genetic lineages within species using wing morphometrics (Akahira & Sakagami, 1959; Carneiro et al., 2019; Francoy et al., 2008, 2011). Fewer studies, however, have investigated population‐level variation in wing venation (Francoy et al., 2011; Rossa et al., 2016). This variation in wing morphology can serve as a useful proxy for assessing the extent of divergence between closely related populations (Oleksa & Tofilski, 2015; Peil & Aranda, 2021). More broadly, these results provide robust evidence for microevolutionary change in wing morphology across reproductively isolated bee populations.

As for many island bee populations, it is unknown how and when this population of *H. tripartitus* colonized Santa Cruz Island. Island dispersal by bees is generally poorly understood, though phylogenetic analyses and behavioral studies can offer clues to potential avenues for colonization events. On Santa Cruz Island, colonization by many bee species could have occurred during the Last Glacial Maximum (17,000–18,000 years ago), when lowered sea levels reduced the distance from the mainland to about 6 km (Miller, 1985). Gene flow across the channel may have continued for an unknown period, depending primarily on the distance of the water barrier and the dispersal capabilities of *H. tripartitus*. Dispersal timing aside, it is evident from our results that the Santa Cruz Island population has diverged morphologically from the mainland population. The unique selective environment of the island (i.e., including climatic and ecological differences from the mainland) may contribute to this population divergence, in addition to founder effect and genetic drift. Future sampling across the entire Channel Island archipelago could shed light on historical patterns of dispersal and population divergence, inferred from patterns of differentiation in wing landmark geometries. Finally, comparisons to other populations across the considerable geographic range of *H. tripartitus* would provide interesting context for assessing the relative magnitude of phenotypic divergence in the Santa Cruz Island population.

These results highlight the utility of geometric morphometrics for quantifying complex patterns of phenotypic variation that elude observation via simple measurement techniques. The application of geometric morphometrics to insect wing venation patterns is still a relatively recent development, but already has shown promise for species identifications (Aytekin et al., 2007; Francoy et al., 2009; Rattanawannee et al., 2010). Our accurate discrimination between three *Halictus* species likewise supports a role for geometric morphometrics in taxonomic identification to the species level. Further, geometric wing morphometrics may be useful for distinguishing among populations (Henriques et al., 2020; Rossa et al., 2016 ) and between species within complexes (Francoy et al., 2011). Identifying features of wing venation have even been successfully integrated into computer‐aided identification systems, which can accurately identify bee specimens to species and even subspecies from images of wings (Buschbacher et al., 2020; Rattanawannee et al., 2012). Our results indicate that population variation in wing venation can be successfully discriminated using geometric morphometrics, and suggest that these patterns could be usefully extended toward automated identification systems with the aim of further classifying specimens to the population level.

Beyond its use in population identification, wing morphometry holds valuable potential for large‐scale population studies, by providing a tractable alternative to more costly and time‐consuming molecular methods for analyzing population structure. Unlike some morphological traits that can degrade over time, wing venation is strongly preserved in museum specimens, presenting opportunities for sampling of existing specimens in place of conducting new surveys. Wings represent powerful candidates for geometric morphometric analysis because their two‐dimensional surfaces lend themselves to straightforward imaging, in contrast to three‐dimensional traits that require additional protocols to standardize orientation within images. Future studies seeking to identify bees to species or population level may find this methodology viable and potentially more adaptable than traditional taxonomic identifications using dichotomous keys. In particular, we envision that wing morphometrics could increase the feasibility of large‐scale monitoring projects by reducing taxonomic labor (Engel et al., 2021).

In conclusion, we demonstrated species‐ and population‐level variation in *Halictus* wing venation. Our results provide evidence for the divergence of wing venation patterns in isolated island and mainland populations of *H. tripartitus*. Our study emphasizes that wing venation patterns can act as quantifiable indicators of phenotypic differentiation within species, and may be useful for inferring the extent of variation among reproductively isolated populations. Morphological population signatures such as these hold enormous potential for enabling broader assessments of evolutionary change across insect populations over time, over geographic space, or with climatic variables.

## AUTHOR CONTRIBUTIONS

**Madeleine M. Ostwald:** Project administration (supporting); supervision (equal); validation (supporting); visualization (equal); writing – original draft (equal); writing – review and editing (lead). **Charles N. Thrift:** Conceptualization (supporting); data curation (lead); formal analysis (lead); funding acquisition (supporting); investigation (lead); methodology (equal); resources (supporting); software (lead); validation (equal); visualization (equal); writing – original draft (equal); writing – review and editing (supporting). **Katja C. Seltmann:** Conceptualization (lead); data curation (supporting); funding acquisition (lead); investigation (supporting); methodology (supporting); project administration (lead); resources (lead); supervision (lead); validation (equal); writing – original draft (supporting); writing – review and editing (supporting).

## Supporting information


Table S1.

Table S2.

Figure S1.

Data S1.

## Data Availability

All data associated with this study are publicly available in Zenodo.
