# Supplementary figures and images for: Phenotypic divergence in an island bee population: Applying geometric morphometrics to discriminate population‐level variation in wing venation
Source: Ecol Evol. 2023 May 10;13(5):e10085. doi: 10.1002/ece3.10085 (PMC10172614; doi:10.1002/ece3.10085)

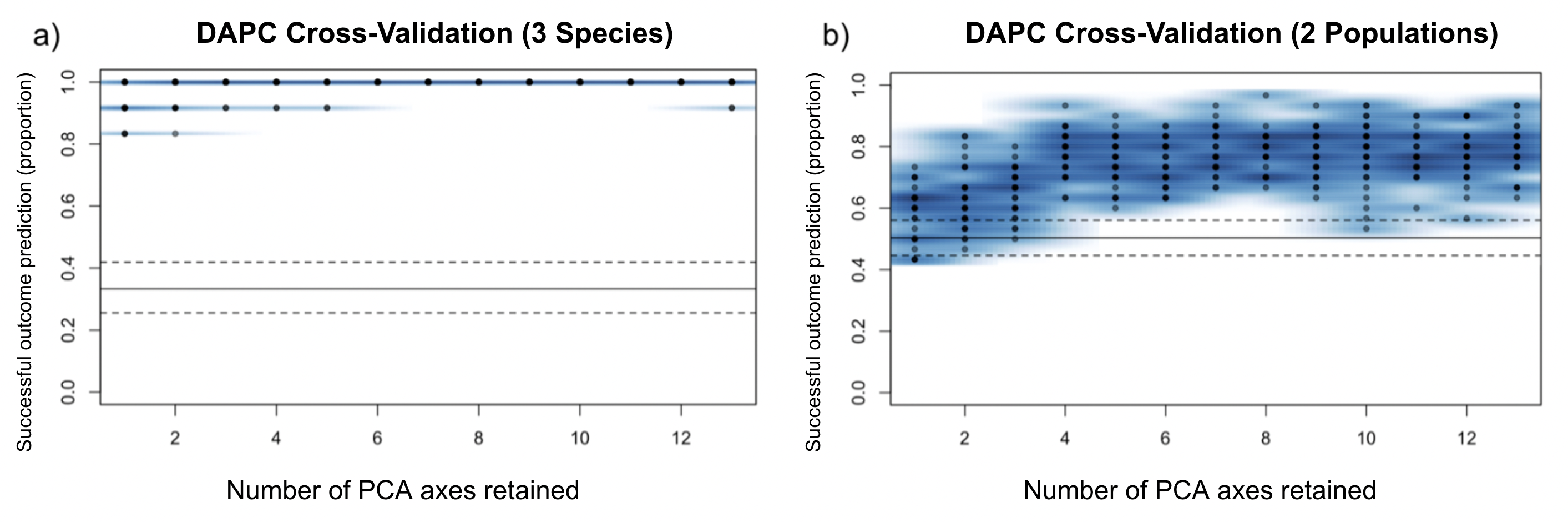

Supplement: Supplementary file 3 — Figure S1. [file ECE3-13-e10085-s002.png]
