# Supplementary material for: Phenotypic divergence in an island bee population: Applying geometric morphometrics to discriminate population‐level variation in wing venation
Source: Ecol Evol. 2023 May 10;13(5):e10085. doi: 10.1002/ece3.10085 (PMC10172614; doi:10.1002/ece3.10085)

**Supplementary Materials**

Supplementary Table 1: Mean landmark coordinate values by species and population.

|  | *Halictus tripartitus* | | *Halictus ligatus* | *Halictus farinosus* |
| --- | --- | --- | --- | --- |
| Landmark | Mainland population | Island population |  |  |
| 1x | 0.394 | 0.392 | 0.395 | 0.386 |
| 1y | 0.153 | 0.153 | 0.127 | 0.136 |
| 2x | 0.242 | 0.244 | 0.267 | 0.292 |
| 2y | 0.256 | 0.253 | 0.249 | 0.275 |
| 3x | -0.025 | -0.031 | -0.032 | -0.024 |
| 3y | 0.185 | 0.185 | 0.181 | 0.177 |
| 4x | -0.121 | -0.122 | -0.119 | -0.113 |
| 4y | 0.206 | 0.207 | 0.202 | 0.197 |
| 5x | -0.321 | -0.320 | -0.339 | -0.338 |
| 5y | 0.010 | 0.014 | 0.005 | -0.005 |
| 6x | -0.488 | -0.488 | -0.503 | -0.496 |
| 6y | -0.025 | -0.023 | -0.036 | -0.046 |
| 7x | 0.030 | 0.032 | 0.029 | 0.024 |
| 7y | -0.175 | -0.176 | -0.159 | -0.163 |
| 8x | 0.100 | 0.106 | 0.109 | 0.093 |
| 8y | -0.290 | -0.293 | -0.271 | -0.274 |
| 9x | 0.188 | 0.187 | 0.193 | 0.175 |
| 9y | -0.321 | -0.320 | -0.298 | -0.297 |

Supplementary Table 2: Catalog numbers for each specimen used, either in population- or species-level analysis.

| catalogNumber | species | population | populationAnalysis? | speciesAnalysis? |
| --- | --- | --- | --- | --- |
| UCSB_IZC00001300 | Halictus ligatus | Mainland | No | Yes |
| UCSB_IZC00003754 | Halictus tripartitus | Santa Cruz Island | Yes | No |
| UCSB_IZC00003760 | Halictus farinosus | Santa Cruz Island | No | Yes |
| UCSB_IZC00004911 | Halictus farinosus | Santa Cruz Island | No | Yes |
| UCSB_IZC00008436 | Halictus tripartitus | Mainland | Yes | No |
| UCSB_IZC00010805 | Halictus tripartitus | Mainland | Yes | No |
| UCSB_IZC00012007 | Halictus tripartitus | Mainland | Yes | No |
| UCSB_IZC00012008 | Halictus tripartitus | Mainland | Yes | No |
| UCSB_IZC00012012 | Halictus tripartitus | Mainland | Yes | Yes |
| UCSB_IZC00012014 | Halictus tripartitus | Mainland | Yes | No |
| UCSB_IZC00012019 | Halictus tripartitus | Mainland | Yes | No |
| UCSB_IZC00012020 | Halictus tripartitus | Mainland | Yes | No |
| UCSB_IZC00012024 | Halictus tripartitus | Mainland | Yes | No |
| UCSB_IZC00012030 | Halictus tripartitus | Mainland | Yes | No |
| UCSB_IZC00012031 | Halictus tripartitus | Mainland | Yes | No |
| UCSB_IZC00012032 | Halictus tripartitus | Mainland | Yes | No |
| UCSB_IZC00012037 | Halictus tripartitus | Mainland | Yes | No |
| UCSB_IZC00012038 | Halictus tripartitus | Mainland | Yes | No |
| UCSB_IZC00012043 | Halictus tripartitus | Mainland | Yes | No |
| UCSB_IZC00012044 | Halictus ligatus | Mainland | No | Yes |
| UCSB_IZC00012048 | Halictus tripartitus | Mainland | Yes | No |
| UCSB_IZC00012055 | Halictus tripartitus | Mainland | Yes | No |
| UCSB_IZC00012060 | Halictus tripartitus | Mainland | Yes | Yes |
| UCSB_IZC00012188 | Halictus tripartitus | Mainland | Yes | No |
| UCSB_IZC00012190 | Halictus tripartitus | Mainland | Yes | Yes |
| UCSB_IZC00012196 | Halictus tripartitus | Mainland | Yes | No |
| UCSB_IZC00012198 | Halictus tripartitus | Mainland | Yes | No |
| UCSB_IZC00012208 | Halictus tripartitus | Mainland | Yes | No |
| UCSB_IZC00012247 | Halictus tripartitus | Mainland | Yes | No |
| UCSB_IZC00012258 | Halictus tripartitus | Mainland | Yes | No |
| UCSB_IZC00012275 | Halictus tripartitus | Mainland | Yes | No |
| UCSB_IZC00012288 | Halictus tripartitus | Mainland | Yes | No |
| UCSB_IZC00012295 | Halictus tripartitus | Mainland | Yes | No |
| UCSB_IZC00012304 | Halictus tripartitus | Mainland | Yes | No |
| UCSB_IZC00012310 | Halictus tripartitus | Mainland | Yes | No |
| UCSB_IZC00012311 | Halictus tripartitus | Mainland | Yes | No |
| UCSB_IZC00012334 | Halictus tripartitus | Mainland | Yes | No |
| UCSB_IZC00012335 | Halictus tripartitus | Mainland | Yes | No |
| UCSB_IZC00012341 | Halictus tripartitus | Mainland | Yes | No |
| UCSB_IZC00012344 | Halictus tripartitus | Mainland | Yes | No |
| UCSB_IZC00012347 | Halictus tripartitus | Mainland | Yes | No |
| UCSB_IZC00012348 | Halictus tripartitus | Mainland | Yes | Yes |
| UCSB_IZC00012350 | Halictus tripartitus | Mainland | Yes | No |
| UCSB_IZC00012353 | Halictus tripartitus | Mainland | Yes | No |
| UCSB_IZC00012354 | Halictus tripartitus | Mainland | Yes | No |
| UCSB_IZC00012357 | Halictus tripartitus | Mainland | Yes | Yes |
| UCSB_IZC00012421 | Halictus tripartitus | Mainland | Yes | No |
| UCSB_IZC00012423 | Halictus tripartitus | Mainland | Yes | Yes |
| UCSB_IZC00012437 | Halictus ligatus | Mainland | No | Yes |
| UCSB_IZC00012439 | Halictus tripartitus | Mainland | Yes | No |
| UCSB_IZC00012441 | Halictus tripartitus | Mainland | Yes | No |
| UCSB_IZC00012443 | Halictus tripartitus | Mainland | Yes | No |
| UCSB_IZC00012451 | Halictus tripartitus | Mainland | Yes | No |
| UCSB_IZC00012458 | Halictus ligatus | Mainland | No | Yes |
| UCSB_IZC00012460 | Halictus tripartitus | Mainland | Yes | No |
| UCSB_IZC00012472 | Halictus tripartitus | Mainland | Yes | No |
| UCSB_IZC00012477 | Halictus tripartitus | Mainland | Yes | No |
| UCSB_IZC00014644 | Halictus tripartitus | Mainland | Yes | No |
| UCSB_IZC00014900 | Halictus tripartitus | Mainland | Yes | No |
| UCSB_IZC00014942 | Halictus tripartitus | Mainland | Yes | No |
| UCSB_IZC00014982 | Halictus tripartitus | Mainland | Yes | No |
| UCSB_IZC00015024 | Halictus tripartitus | Mainland | Yes | No |
| UCSB_IZC00015024 | Halictus tripartitus | Mainland | No | Yes |
| UCSB_IZC00015026 | Halictus tripartitus | Mainland | Yes | No |
| UCSB_IZC00015151 | Halictus tripartitus | Mainland | Yes | Yes |
| UCSB_IZC00015180 | Halictus tripartitus | Mainland | Yes | No |
| UCSB_IZC00015229 | Halictus tripartitus | Mainland | Yes | No |
| UCSB_IZC00022218 | Halictus tripartitus | Mainland | Yes | No |
| UCSB_IZC00022219 | Halictus tripartitus | Mainland | Yes | No |
| UCSB_IZC00028329 | Halictus tripartitus | Mainland | Yes | No |
| UCSB_IZC00028368 | Halictus tripartitus | Mainland | Yes | Yes |
| UCSB_IZC00028538 | Halictus tripartitus | Mainland | No | Yes |
| UCSB_IZC00028854 | Halictus ligatus | Mainland | No | Yes |
| UCSB_IZC00028872 | Halictus ligatus | Mainland | No | Yes |
| UCSB_IZC00028900 | Halictus tripartitus | Mainland | Yes | No |
| UCSB_IZC00030014 | Halictus tripartitus | Santa Cruz Island | Yes | No |
| UCSB_IZC00030018 | Halictus tripartitus | Santa Cruz Island | Yes | Yes |
| UCSB_IZC00030019 | Halictus tripartitus | Santa Cruz Island | Yes | No |
| UCSB_IZC00030022 | Halictus tripartitus | Mainland | Yes | No |
| UCSB_IZC00030025 | Halictus tripartitus | Mainland | Yes | No |
| UCSB_IZC00030060 | Halictus tripartitus | Santa Cruz Island | Yes | No |
| UCSB_IZC00030063 | Halictus tripartitus | Santa Cruz Island | Yes | No |
| UCSB_IZC00030067 | Halictus tripartitus | Santa Cruz Island | Yes | No |
| UCSB_IZC00030073 | Halictus tripartitus | Mainland | Yes | Yes |
| UCSB_IZC00030095 | Halictus tripartitus | Santa Cruz Island | Yes | No |
| UCSB_IZC00030096 | Halictus tripartitus | Mainland | Yes | No |
| UCSB_IZC00030111 | Halictus tripartitus | Santa Cruz Island | Yes | No |
| UCSB_IZC00030113 | Halictus tripartitus | Santa Cruz Island | Yes | No |
| UCSB_IZC00030121 | Halictus farinosus | Santa Cruz Island | No | Yes |
| UCSB_IZC00030122 | Halictus farinosus | Santa Cruz Island | No | Yes |
| UCSB_IZC00030123 | Halictus tripartitus | Mainland | Yes | No |
| UCSB_IZC00030127 | Halictus tripartitus | Santa Cruz Island | Yes | No |
| UCSB_IZC00030129 | Halictus ligatus | Mainland | No | Yes |
| UCSB_IZC00030131 | Halictus tripartitus | Santa Cruz Island | Yes | No |
| UCSB_IZC00030139 | Halictus tripartitus | Santa Cruz Island | Yes | Yes |
| UCSB_IZC00030140 | Halictus tripartitus | Santa Cruz Island | Yes | No |
| UCSB_IZC00030146 | Halictus tripartitus | Santa Cruz Island | Yes | Yes |
| UCSB_IZC00030155 | Halictus tripartitus | Santa Cruz Island | Yes | No |
| UCSB_IZC00030158 | Halictus tripartitus | Mainland | Yes | No |
| UCSB_IZC00030166 | Halictus tripartitus | Mainland | Yes | No |
| UCSB_IZC00030168 | Halictus tripartitus | Santa Cruz Island | Yes | No |
| UCSB_IZC00030174 | Halictus tripartitus | Santa Cruz Island | Yes | No |
| UCSB_IZC00030194 | Halictus tripartitus | Mainland | Yes | Yes |
| UCSB_IZC00030209 | Halictus tripartitus | Santa Cruz Island | Yes | Yes |
| UCSB_IZC00030212 | Halictus tripartitus | Santa Cruz Island | Yes | No |
| UCSB_IZC00030229 | Halictus tripartitus | Santa Cruz Island | Yes | No |
| UCSB_IZC00030248 | Halictus tripartitus | Mainland | Yes | No |
| UCSB_IZC00030263 | Halictus tripartitus | Santa Cruz Island | Yes | No |
| UCSB_IZC00030265 | Halictus tripartitus | Mainland | Yes | No |
| UCSB_IZC00030269 | Halictus ligatus | Mainland | No | Yes |
| UCSB_IZC00030275 | Halictus tripartitus | Santa Cruz Island | Yes | No |
| UCSB_IZC00030277 | Halictus tripartitus | Santa Cruz Island | Yes | No |
| UCSB_IZC00030303 | Halictus tripartitus | Santa Cruz Island | Yes | Yes |
| UCSB_IZC00030320 | Halictus tripartitus | Mainland | Yes | Yes |
| UCSB_IZC00030326 | Halictus tripartitus | Mainland | Yes | No |
| UCSB_IZC00030330 | Halictus tripartitus | Mainland | Yes | No |
| UCSB_IZC00030336 | Halictus tripartitus | Santa Cruz Island | Yes | No |
| UCSB_IZC00030348 | Halictus farinosus | Santa Cruz Island | No | Yes |
| UCSB_IZC00030349 | Halictus tripartitus | Santa Cruz Island | Yes | No |
| UCSB_IZC00030350 | Halictus tripartitus | Santa Cruz Island | Yes | No |
| UCSB_IZC00030351 | Halictus tripartitus | Mainland | Yes | No |
| UCSB_IZC00030355 | Halictus tripartitus | Mainland | Yes | No |
| UCSB_IZC00030373 | Halictus tripartitus | Santa Cruz Island | Yes | No |
| UCSB_IZC00030374 | Halictus tripartitus | Mainland | Yes | No |
| UCSB_IZC00030381 | Halictus tripartitus | Mainland | Yes | No |
| UCSB_IZC00030382 | Halictus tripartitus | Santa Cruz Island | Yes | No |
| UCSB_IZC00030392 | Halictus tripartitus | Santa Cruz Island | Yes | No |
| UCSB_IZC00030397 | Halictus tripartitus | Mainland | Yes | No |
| UCSB_IZC00030405 | Halictus tripartitus | Santa Cruz Island | Yes | No |
| UCSB_IZC00030414 | Halictus tripartitus | Mainland | Yes | No |
| UCSB_IZC00030439 | Halictus tripartitus | Mainland | Yes | No |
| UCSB_IZC00030442 | Halictus tripartitus | Santa Cruz Island | Yes | No |
| UCSB_IZC00030444 | Halictus tripartitus | Santa Cruz Island | Yes | No |
| UCSB_IZC00030451 | Halictus tripartitus | Mainland | Yes | Yes |
| UCSB_IZC00030461 | Halictus tripartitus | Santa Cruz Island | Yes | No |
| UCSB_IZC00030463 | Halictus tripartitus | Santa Cruz Island | Yes | No |
| UCSB_IZC00030467 | Halictus tripartitus | Santa Cruz Island | Yes | No |
| UCSB_IZC00030468 | Halictus tripartitus | Mainland | Yes | No |
| UCSB_IZC00030472 | Halictus tripartitus | Mainland | Yes | Yes |
| UCSB_IZC00030486 | Halictus tripartitus | Mainland | Yes | No |
| UCSB_IZC00030496 | Halictus tripartitus | Mainland | Yes | No |
| UCSB_IZC00030506 | Halictus tripartitus | Santa Cruz Island | Yes | No |
| UCSB_IZC00030508 | Halictus tripartitus | Mainland | Yes | Yes |
| UCSB_IZC00030520 | Halictus tripartitus | Mainland | Yes | No |
| UCSB_IZC00030539 | Halictus tripartitus | Santa Cruz Island | Yes | No |
| UCSB_IZC00030540 | Halictus tripartitus | Santa Cruz Island | Yes | No |
| UCSB_IZC00030701 | Halictus tripartitus | Santa Cruz Island | Yes | No |
| UCSB_IZC00030780 | Halictus tripartitus | Santa Cruz Island | Yes | No |
| UCSB_IZC00030912 | Halictus tripartitus | Mainland | Yes | No |
| UCSB_IZC00030951 | Halictus tripartitus | Santa Cruz Island | Yes | No |
| UCSB_IZC00031075 | Halictus tripartitus | Santa Cruz Island | Yes | No |
| UCSB_IZC00031103 | Halictus tripartitus | Mainland | Yes | No |
| UCSB_IZC00033295 | Halictus tripartitus | Santa Cruz Island | Yes | Yes |
| UCSB_IZC00034036 | Halictus tripartitus | Santa Cruz Island | Yes | No |
| UCSB_IZC00034038 | Halictus ligatus | Mainland | No | Yes |
| UCSB_IZC00034049 | Halictus tripartitus | Mainland | Yes | No |
| UCSB_IZC00034060 | Halictus tripartitus | Santa Cruz Island | Yes | No |
| UCSB_IZC00034080 | Halictus tripartitus | Mainland | Yes | No |
| UCSB_IZC00034513 | Halictus farinosus | Mainland | No | Yes |
| UCSB_IZC00034574 | Halictus tripartitus | Santa Cruz Island | Yes | No |
| UCSB_IZC00034592 | Halictus ligatus | Mainland | No | Yes |
| UCSB_IZC00034598 | Halictus tripartitus | Mainland | Yes | No |
| UCSB_IZC00034616 | Halictus tripartitus | Santa Cruz Island | Yes | No |
| UCSB_IZC00034799 | Halictus farinosus | Santa Cruz Island | No | Yes |
| UCSB_IZC00034801 | Halictus ligatus | Mainland | No | Yes |
| UCSB_IZC00034809 | Halictus ligatus | Mainland | No | Yes |
| UCSB_IZC00035244 | Halictus tripartitus | Mainland | Yes | No |
| UCSB_IZC00035255 | Halictus tripartitus | Santa Cruz Island | Yes | No |
| UCSB_IZC00035279 | Halictus tripartitus | Mainland | Yes | No |
| UCSB_IZC00035287 | Halictus tripartitus | Mainland | Yes | No |
| UCSB_IZC00035317 | Halictus tripartitus | Mainland | Yes | No |
| UCSB_IZC00035328 | Halictus tripartitus | Santa Cruz Island | Yes | No |
| UCSB_IZC00035331 | Halictus tripartitus | Mainland | Yes | No |
| UCSB_IZC00035335 | Halictus tripartitus | Mainland | Yes | No |
| UCSB_IZC00035446 | Halictus tripartitus | Mainland | Yes | Yes |
| UCSB_IZC00035447 | Halictus tripartitus | Santa Cruz Island | Yes | No |
| UCSB_IZC00035501 | Halictus tripartitus | Mainland | Yes | Yes |
| UCSB_IZC00035550 | Halictus tripartitus | Mainland | Yes | No |
| UCSB_IZC00035556 | Halictus tripartitus | Mainland | Yes | No |
| UCSB_IZC00035659 | Halictus tripartitus | Mainland | Yes | No |
| UCSB_IZC00035841 | Halictus ligatus | Mainland | No | Yes |
| UCSB_IZC00035950 | Halictus ligatus | Mainland | No | Yes |
| UCSB_IZC00036006 | Halictus ligatus | Mainland | No | Yes |
| UCSB_IZC00036124 | Halictus tripartitus | Mainland | Yes | No |
| UCSB_IZC00036175 | Halictus tripartitus | Mainland | Yes | No |
| UCSB_IZC00036334 | Halictus ligatus | Mainland | No | Yes |
| UCSB_IZC00036335 | Halictus ligatus | Mainland | No | Yes |
| UCSB_IZC00036500 | Halictus tripartitus | Santa Cruz Island | Yes | Yes |
| UCSB_IZC00036721 | Halictus farinosus | Santa Cruz Island | No | Yes |
| UCSB_IZC00036722 | Halictus farinosus | Santa Cruz Island | No | Yes |
| UCSB_IZC00036732 | Halictus tripartitus | Santa Cruz Island | Yes | No |
| UCSB_IZC00036735 | Halictus tripartitus | Santa Cruz Island | Yes | No |
| UCSB_IZC00036739 | Halictus tripartitus | Santa Cruz Island | Yes | No |
| UCSB_IZC00036745 | Halictus farinosus | Santa Cruz Island | No | Yes |
| UCSB_IZC00036750 | Halictus tripartitus | Santa Cruz Island | Yes | No |
| UCSB_IZC00036764 | Halictus tripartitus | Santa Cruz Island | Yes | No |
| UCSB_IZC00036767 | Halictus farinosus | Santa Cruz Island | No | Yes |
| UCSB_IZC00036774 | Halictus tripartitus | Santa Cruz Island | Yes | No |
| UCSB_IZC00036777 | Halictus tripartitus | Santa Cruz Island | Yes | No |
| UCSB_IZC00036835 | Halictus farinosus | Santa Cruz Island | No | Yes |
| UCSB_IZC00036852 | Halictus farinosus | Santa Cruz Island | No | Yes |
| UCSB_IZC00036854 | Halictus farinosus | Santa Cruz Island | No | Yes |
| UCSB_IZC00036860 | Halictus farinosus | Santa Cruz Island | No | Yes |
| UCSB_IZC00036862 | Halictus farinosus | Santa Cruz Island | No | Yes |
| UCSB_IZC00036864 | Halictus farinosus | Santa Cruz Island | No | Yes |
| UCSB_IZC00036870 | Halictus ligatus | Mainland | No | Yes |
| UCSB_IZC00036873 | Halictus ligatus | Mainland | No | Yes |
| UCSB_IZC00036879 | Halictus farinosus | Santa Cruz Island | No | Yes |
| UCSB_IZC00036903 | Halictus farinosus | Santa Cruz Island | No | Yes |
| UCSB_IZC00036908 | Halictus farinosus | Santa Cruz Island | No | Yes |
| UCSB_IZC00036910 | Halictus tripartitus | Santa Cruz Island | Yes | No |
| UCSB_IZC00036917 | Halictus tripartitus | Santa Cruz Island | Yes | No |
| UCSB_IZC00036944 | Halictus farinosus | Santa Cruz Island | No | Yes |
| UCSB_IZC00036945 | Halictus tripartitus | Santa Cruz Island | Yes | No |
| UCSB_IZC00036955 | Halictus farinosus | Santa Cruz Island | No | Yes |
| UCSB_IZC00036964 | Halictus tripartitus | Santa Cruz Island | Yes | No |
| UCSB_IZC00036968 | Halictus tripartitus | Santa Cruz Island | Yes | No |
| UCSB_IZC00036971 | Halictus tripartitus | Santa Cruz Island | Yes | No |
| UCSB_IZC00036976 | Halictus tripartitus | Santa Cruz Island | Yes | No |
| UCSB_IZC00036993 | Halictus tripartitus | Mainland | Yes | No |
| UCSB_IZC00037003 | Halictus tripartitus | Mainland | Yes | No |
| UCSB_IZC00037020 | Halictus tripartitus | Mainland | Yes | No |
| UCSB_IZC00037027 | Halictus ligatus | Mainland | No | Yes |
| UCSB_IZC00037030 | Halictus farinosus | Santa Cruz Island | No | Yes |
| UCSB_IZC00037036 | Halictus farinosus | Santa Cruz Island | No | Yes |
| UCSB_IZC00037063 | Halictus farinosus | Santa Cruz Island | No | Yes |
| UCSB_IZC00037064 | Halictus tripartitus | Mainland | Yes | No |
| UCSB_IZC00037066 | Halictus farinosus | Santa Cruz Island | No | Yes |
| UCSB_IZC00037072 | Halictus tripartitus | Mainland | Yes | No |
| UCSB_IZC00037074 | Halictus tripartitus | Mainland | Yes | Yes |
| UCSB_IZC00037108 | Halictus farinosus | Santa Cruz Island | No | Yes |
| UCSB_IZC00037110 | Halictus tripartitus | Santa Cruz Island | Yes | No |
| UCSB_IZC00037117 | Halictus farinosus | Santa Cruz Island | No | Yes |
| UCSB_IZC00037126 | Halictus farinosus | Santa Cruz Island | No | Yes |
| UCSB_IZC00037137 | Halictus farinosus | Santa Cruz Island | No | Yes |
| UCSB_IZC00037266 | Halictus tripartitus | Santa Cruz Island | Yes | No |
| UCSB_IZC00037267 | Halictus tripartitus | Santa Cruz Island | Yes | No |
| UCSB_IZC00037281 | Halictus tripartitus | Santa Cruz Island | Yes | No |
| UCSB_IZC00037283 | Halictus tripartitus | Santa Cruz Island | Yes | Yes |
| UCSB_IZC00037285 | Halictus tripartitus | Santa Cruz Island | Yes | No |
| UCSB_IZC00037290 | Halictus tripartitus | Santa Cruz Island | Yes | No |
| UCSB_IZC00037302 | Halictus tripartitus | Santa Cruz Island | Yes | No |
| UCSB_IZC00037317 | Halictus tripartitus | Santa Cruz Island | Yes | No |
| UCSB_IZC00037321 | Halictus tripartitus | Mainland | Yes | No |
| UCSB_IZC00037323 | Halictus tripartitus | Mainland | Yes | No |
| UCSB_IZC00037325 | Halictus tripartitus | Mainland | Yes | No |
| UCSB_IZC00037327 | Halictus tripartitus | Mainland | Yes | Yes |
| UCSB_IZC00037328 | Halictus tripartitus | Mainland | Yes | No |
| UCSB_IZC00037331 | Halictus tripartitus | Mainland | Yes | Yes |
| UCSB_IZC00037342 | Halictus tripartitus | Mainland | Yes | No |
| UCSB_IZC00037348 | Halictus tripartitus | Mainland | Yes | No |
| UCSB_IZC00037351 | Halictus tripartitus | Mainland | Yes | No |
| UCSB_IZC00037367 | Halictus tripartitus | Mainland | Yes | No |
| UCSB_IZC00037368 | Halictus tripartitus | Mainland | Yes | No |
| UCSB_IZC00037371 | Halictus tripartitus | Mainland | Yes | Yes |
| UCSB_IZC00037374 | Halictus tripartitus | Mainland | Yes | No |
| UCSB_IZC00037375 | Halictus tripartitus | Mainland | Yes | No |
| UCSB_IZC00037384 | Halictus tripartitus | Mainland | Yes | Yes |
| UCSB_IZC00037385 | Halictus tripartitus | Mainland | Yes | No |
| UCSB_IZC00037411 | Halictus tripartitus | Mainland | Yes | No |
| UCSB_IZC00037415 | Halictus tripartitus | Mainland | Yes | No |
| UCSB_IZC00037416 | Halictus tripartitus | Mainland | Yes | No |
| UCSB_IZC00037421 | Halictus tripartitus | Mainland | Yes | No |
| UCSB_IZC00037422 | Halictus farinosus | Santa Cruz Island | No | Yes |
| UCSB_IZC00037424 | Halictus tripartitus | Mainland | Yes | No |
| UCSB_IZC00037429 | Halictus tripartitus | Mainland | Yes | No |
| UCSB_IZC00037430 | Halictus tripartitus | Mainland | Yes | No |
| UCSB_IZC00037437 | Halictus tripartitus | Mainland | Yes | No |
| UCSB_IZC00037440 | Halictus tripartitus | Mainland | Yes | No |
| UCSB_IZC00037449 | Halictus tripartitus | Mainland | Yes | No |
| UCSB_IZC00037450 | Halictus tripartitus | Mainland | Yes | No |
| UCSB_IZC00037451 | Halictus ligatus | Mainland | No | Yes |
| UCSB_IZC00037457 | Halictus tripartitus | Mainland | Yes | No |
| UCSB_IZC00037463 | Halictus tripartitus | Mainland | Yes | Yes |
| UCSB_IZC00037475 | Halictus tripartitus | Mainland | Yes | No |
| UCSB_IZC00037485 | Halictus tripartitus | Mainland | Yes | No |
| UCSB_IZC00037490 | Halictus tripartitus | Mainland | Yes | Yes |
| UCSB_IZC00037497 | Halictus tripartitus | Mainland | Yes | No |
| UCSB_IZC00037696 | Halictus ligatus | Mainland | No | Yes |
| UCSB_IZC00037732 | Halictus ligatus | Mainland | No | Yes |
| UCSB_IZC00038802 | Halictus tripartitus | Santa Cruz Island | Yes | Yes |
| UCSB_IZC00038851 | Halictus tripartitus | Santa Cruz Island | Yes | Yes |
| UCSB_IZC00038912 | Halictus tripartitus | Santa Cruz Island | Yes | No |
| UCSB_IZC00039104 | Halictus tripartitus | Santa Cruz Island | Yes | No |
| UCSB_IZC00039113 | Halictus tripartitus | Santa Cruz Island | Yes | No |
| UCSB_IZC00039120 | Halictus ligatus | Mainland | No | Yes |
| UCSB_IZC00039264 | Halictus farinosus | Santa Cruz Island | No | Yes |
| UCSB_IZC00039336 | Halictus farinosus | Santa Cruz Island | No | Yes |
| UCSB_IZC00039363 | Halictus tripartitus | Santa Cruz Island | Yes | No |
| UCSB_IZC00039378 | Halictus tripartitus | Santa Cruz Island | Yes | No |
| UCSB_IZC00039390 | Halictus tripartitus | Santa Cruz Island | Yes | No |
| UCSB_IZC00039392 | Halictus ligatus | Mainland | No | Yes |
| UCSB_IZC00039403 | Halictus tripartitus | Santa Cruz Island | Yes | No |
| UCSB_IZC00039404 | Halictus farinosus | Santa Cruz Island | No | Yes |
| UCSB_IZC00039427 | Halictus tripartitus | Santa Cruz Island | Yes | No |
| UCSB_IZC00039438 | Halictus tripartitus | Santa Cruz Island | Yes | No |
| UCSB_IZC00039441 | Halictus tripartitus | Santa Cruz Island | Yes | No |
| UCSB_IZC00039475 | Halictus tripartitus | Santa Cruz Island | Yes | Yes |
| UCSB_IZC00039519 | Halictus tripartitus | Santa Cruz Island | Yes | No |
| UCSB_IZC00039522 | Halictus farinosus | Santa Cruz Island | No | Yes |
| UCSB_IZC00039536 | Halictus farinosus | Santa Cruz Island | No | Yes |
| UCSB_IZC00039543 | Halictus tripartitus | Santa Cruz Island | Yes | No |
| UCSB_IZC00039566 | Halictus tripartitus | Santa Cruz Island | Yes | Yes |
| UCSB_IZC00039602 | Halictus ligatus | Mainland | No | Yes |
| UCSB_IZC00039613 | Halictus tripartitus | Santa Cruz Island | Yes | No |
| UCSB_IZC00039649 | Halictus tripartitus | Santa Cruz Island | Yes | No |
| UCSB_IZC00039828 | Halictus tripartitus | Santa Cruz Island | Yes | No |
| UCSB_IZC00039848 | Halictus tripartitus | Santa Cruz Island | Yes | No |
| UCSB_IZC00039861 | Halictus tripartitus | Santa Cruz Island | Yes | No |
| UCSB_IZC00039867 | Halictus tripartitus | Santa Cruz Island | Yes | No |
| UCSB_IZC00039880 | Halictus tripartitus | Santa Cruz Island | Yes | No |
| UCSB_IZC00039888 | Halictus tripartitus | Santa Cruz Island | Yes | No |
| UCSB_IZC00039889 | Halictus farinosus | Santa Cruz Island | No | Yes |
| UCSB_IZC00039890 | Halictus tripartitus | Santa Cruz Island | Yes | No |
| UCSB_IZC00039910 | Halictus tripartitus | Santa Cruz Island | Yes | No |
| UCSB_IZC00039912 | Halictus tripartitus | Santa Cruz Island | Yes | No |
| UCSB_IZC00039916 | Halictus tripartitus | Santa Cruz Island | Yes | No |
| UCSB_IZC00039926 | Halictus tripartitus | Santa Cruz Island | Yes | No |
| UCSB_IZC00039933 | Halictus farinosus | Santa Cruz Island | No | Yes |
| UCSB_IZC00039939 | Halictus tripartitus | Santa Cruz Island | Yes | No |
| UCSB_IZC00039960 | Halictus farinosus | Santa Cruz Island | No | Yes |
| UCSB_IZC00040275 | Halictus tripartitus | Santa Cruz Island | Yes | No |
| UCSB_IZC00040281 | Halictus tripartitus | Santa Cruz Island | Yes | No |
| UCSB_IZC00040289 | Halictus tripartitus | Santa Cruz Island | Yes | No |
| UCSB_IZC00040292 | Halictus tripartitus | Santa Cruz Island | Yes | No |
| UCSB_IZC00040298 | Halictus tripartitus | Santa Cruz Island | Yes | Yes |
| UCSB_IZC00040305 | Halictus tripartitus | Santa Cruz Island | Yes | No |
| UCSB_IZC00040420 | Halictus tripartitus | Santa Cruz Island | Yes | No |
| UCSB_IZC00040430 | Halictus tripartitus | Santa Cruz Island | Yes | Yes |
| UCSB_IZC00040916 | Halictus farinosus | Mainland | No | Yes |
| UCSB_IZC00040919 | Halictus ligatus | Mainland | No | Yes |
| UCSB_IZC00040997 | Halictus ligatus | Mainland | No | Yes |
| UCSB_IZC00041012 | Halictus farinosus | Mainland | No | Yes |
| UCSB_IZC00041484 | Halictus ligatus | Mainland | No | Yes |
| UCSB_IZC00041545 | Halictus tripartitus | Santa Cruz Island | Yes | Yes |
| UCSB_IZC00041647 | Halictus ligatus | Mainland | No | Yes |
| UCSB_IZC00041650 | Halictus ligatus | Mainland | No | Yes |
| UCSB_IZC00041659 | Halictus ligatus | Mainland | No | Yes |
| UCSB_IZC00041680 | Halictus ligatus | Mainland | No | Yes |
| UCSB_IZC00041825 | Halictus tripartitus | Santa Cruz Island | Yes | No |
| UCSB_IZC00041831 | Halictus tripartitus | Santa Cruz Island | Yes | No |
| UCSB_IZC00041854 | Halictus tripartitus | Santa Cruz Island | Yes | No |
| UCSB_IZC00041864 | Halictus tripartitus | Santa Cruz Island | Yes | No |
| UCSB_IZC00041868 | Halictus tripartitus | Santa Cruz Island | Yes | No |
| UCSB_IZC00041871 | Halictus tripartitus | Santa Cruz Island | Yes | No |
| UCSB_IZC00041945 | Halictus ligatus | Mainland | No | Yes |
| UCSB_IZC00042070 | Halictus tripartitus | Santa Cruz Island | Yes | No |
| UCSB_IZC00042100 | Halictus tripartitus | Santa Cruz Island | Yes | No |
| UCSB_IZC00042115 | Halictus tripartitus | Santa Cruz Island | Yes | No |
| UCSB_IZC00042255 | Halictus tripartitus | Santa Cruz Island | Yes | No |
| UCSB_IZC00042287 | Halictus tripartitus | Santa Cruz Island | Yes | No |
| UCSB_IZC00042304 | Halictus farinosus | Santa Cruz Island | No | Yes |
| UCSB_IZC00042339 | Halictus farinosus | Santa Cruz Island | No | Yes |
| UCSB_IZC00042352 | Halictus tripartitus | Santa Cruz Island | Yes | No |
| UCSB_IZC00042354 | Halictus ligatus | Mainland | No | Yes |
| UCSB_IZC00042355 | Halictus tripartitus | Santa Cruz Island | Yes | No |
| UCSB_IZC00042367 | Halictus ligatus | Mainland | No | Yes |
| UCSB_IZC00042376 | Halictus ligatus | Mainland | No | Yes |
| UCSB_IZC00042470 | Halictus ligatus | Mainland | No | Yes |
| UCSB_IZC00042514 | Halictus ligatus | Mainland | No | Yes |
| UCSB_IZC00042565 | Halictus tripartitus | Santa Cruz Island | Yes | No |
| UCSB_IZC00042595 | Halictus tripartitus | Santa Cruz Island | Yes | No |
| UCSB_IZC00042642 | Halictus ligatus | Mainland | No | Yes |
| UCSB_IZC00042653 | Halictus ligatus | Mainland | No | Yes |
| UCSB_IZC00042720 | Halictus ligatus | Mainland | No | Yes |
| UCSB_IZC00042763 | Halictus tripartitus | Santa Cruz Island | Yes | No |
| UCSB_IZC00042780 | Halictus ligatus | Mainland | No | Yes |
| UCSB_IZC00042836 | Halictus tripartitus | Santa Cruz Island | Yes | No |
| UCSB_IZC00042844 | Halictus tripartitus | Santa Cruz Island | Yes | No |
| UCSB_IZC00042847 | Halictus tripartitus | Santa Cruz Island | Yes | No |
| UCSB_IZC00042855 | Halictus tripartitus | Santa Cruz Island | Yes | Yes |
| UCSB_IZC00042863 | Halictus tripartitus | Santa Cruz Island | Yes | No |
| UCSB_IZC00042875 | Halictus tripartitus | Santa Cruz Island | Yes | No |
| UCSB_IZC00042887 | Halictus tripartitus | Santa Cruz Island | Yes | No |
| UCSB_IZC00042889 | Halictus tripartitus | Santa Cruz Island | Yes | Yes |
| UCSB_IZC00042902 | Halictus tripartitus | Santa Cruz Island | Yes | No |
| UCSB_IZC00042907 | Halictus tripartitus | Santa Cruz Island | Yes | Yes |
| UCSB_IZC00042925 | Halictus tripartitus | Santa Cruz Island | Yes | No |
| UCSB_IZC00042937 | Halictus tripartitus | Santa Cruz Island | Yes | No |
| UCSB_IZC00042940 | Halictus tripartitus | Santa Cruz Island | Yes | No |
| UCSB_IZC00042944 | Halictus tripartitus | Santa Cruz Island | Yes | No |
| UCSB_IZC00042945 | Halictus tripartitus | Santa Cruz Island | Yes | Yes |
| UCSB_IZC00042953 | Halictus tripartitus | Santa Cruz Island | Yes | No |
| UCSB_IZC00043011 | Halictus tripartitus | Santa Cruz Island | Yes | No |
| UCSB_IZC00043015 | Halictus tripartitus | Santa Cruz Island | Yes | No |

Supplementary Figure 1: DAPC Cross-Validation plots for a) three congeneric species of *Halictus* and b) two populations of *H. tripartitus*


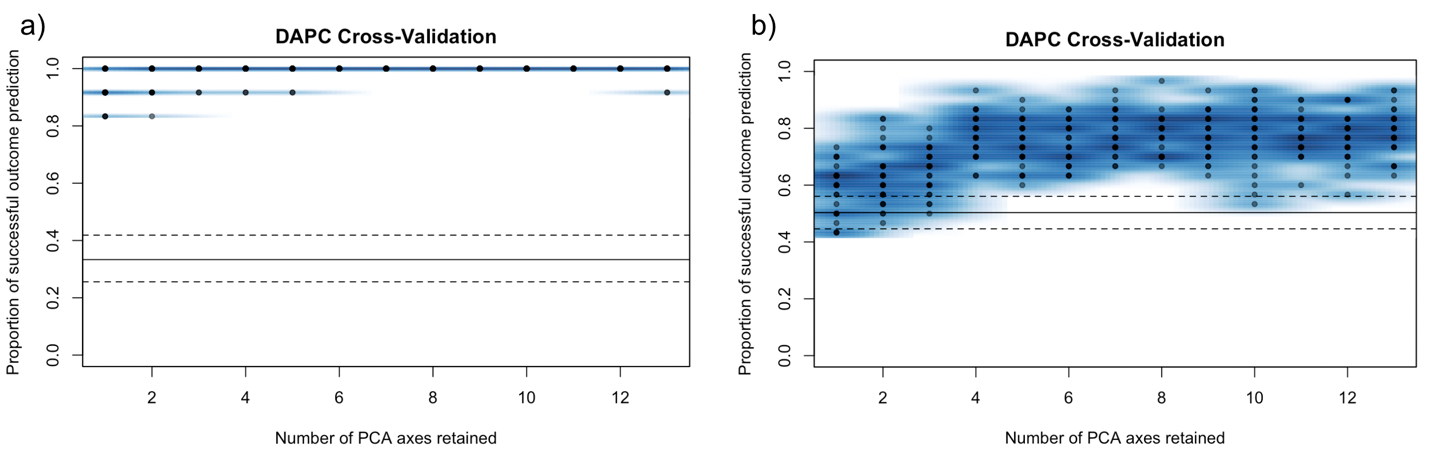

Supplement: Supplementary file 4 — Data S1. [file ECE3-13-e10085-s004.docx]
